# Supplementary material for: Relationship between rheology and structure of interpenetrating, deforming and compressing microgels
Source: Nat Commun. 2019 Jun 4;10:2436. doi: 10.1038/s41467-019-10181-5 (PMC6547648; doi:10.1038/s41467-019-10181-5)
Supplement: Supplementary file 1 — Supplementary Information [file 41467_2019_10181_MOESM1_ESM.pdf]

# – Supplementary Information –

Relationship between Rheology and Structure of Interpenetrating, Deforming and Compressing Microgels

Scheffold et al.

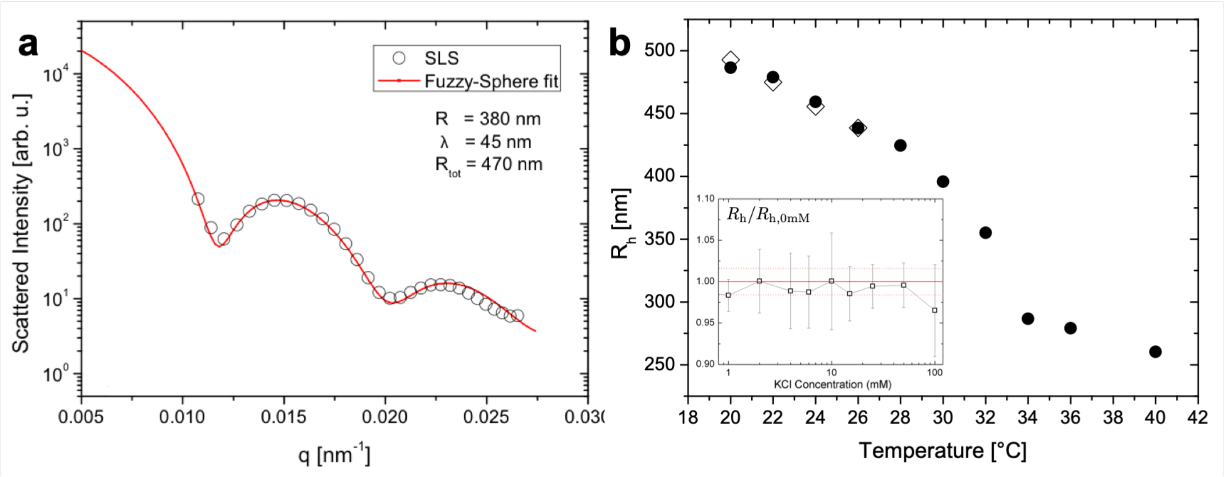

FIG. 1. **Microgel particle size characterization** (a) Static light scattering form factor of a dilute pNIPAM microgel suspension in water. Solid line: Fit with the fuzzy sphere model [1], fitting parameters are $R = 380$ nm for the core radius and $2\sigma = 90$nm for the fuzzy, brush-like, corona. This suggests $R_{\rm tot} = 470$nm and $R/R_{tot} = 0.81$. The size polydispersity parameter used for a best fit to the data is 6%. (b) Microgel volume phase transition (VPT). Temperature dependence of the hydrodynamic radius R_h of pNIPAM microgels in water (from dynamic light scattering, DLS) (•), as used for the rheology study and with 50mM MEA(⋄) added for superresolution imaging. Inset: (◦) Variation of the hydrodynamic radius with added KCl at $T = 22°$C. The red solid line indicates the reference value taken in pure water and the shaded area the corresponding experimental error.

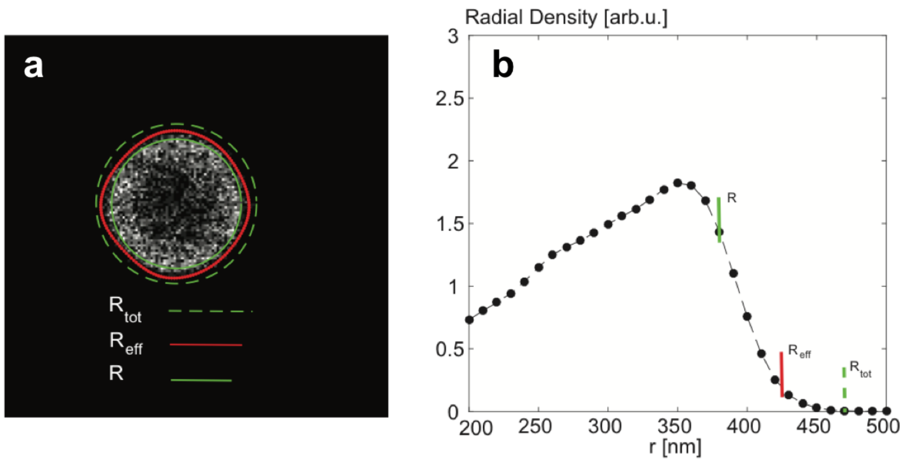

FIG. 2. **dSTORM image of an isolated microgel particle**. (a) The red solid line delineates the microgel boundaries obtained by the Laplacian of Gaussian edge detection method [2], see methods. From the surface area we can derive the effective radius $R_{\rm eff}$ of a corresponding disk shown in Supplementary Figure 5 a) . The dashed line indicates the microgel size including the low density corona with radius $R_{\rm tot} = 470$nm as determined from light scattering, see also Figure 2. (b) Radial density profile of the same microgel particle. Arrows indicate the position of $R = 380$nm, $R_{\rm eff} = 430$nm and $R_{\rm eff} = 470$nm. The reduction of signal towards the center of the particles is due to the labelling protocol (see methods section), which improves contrast but is otherwise insignificant for our analysis.

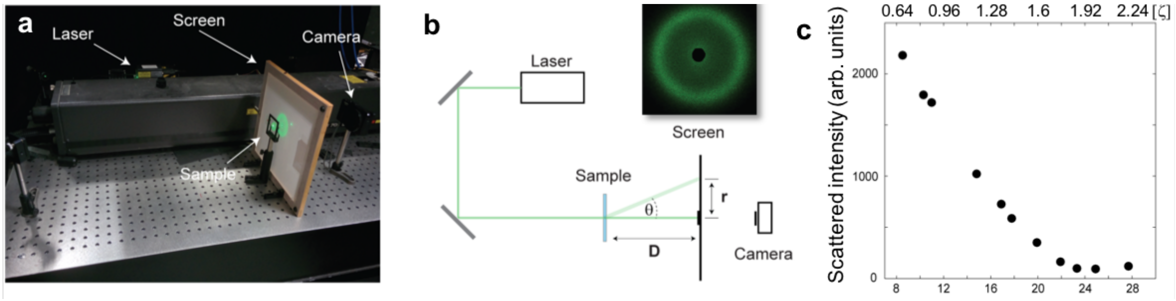

FIG. 3. **Small angle light scattering (SALS) of concentrated microgel suspensions**. (a) Experimental setup: A green laser (Coherent Verdi V5, $\lambda = 532$nm) is used to illuminate the sample as contained in the microscopy sample cell, thickness $L \sim 100 - 200\mu$m. A characteristic diffraction pattern obtained for a dense microgel suspension is visible on the screen placed on the right hand side of the cell. (b) Schematic representation of the setup. This inset shows a scattering pattern recorded at 11 wt% ($\zeta = 0.88$). (c) Total intensity of scattered light obtained by integrating the radially averaged scattered intensity $I(q)$ over the interval $q \in [4/\mu m, 14/\mu m]$, for details see ref. [2].

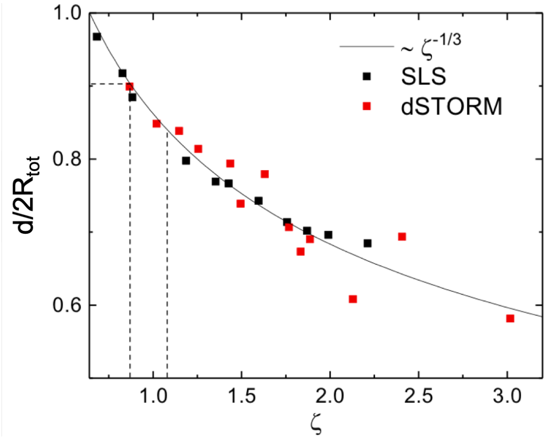

FIG. 4. **Relevant distances upon densely packing the soft microgel particles**. Center-to-center distances obtained experimentally from the position of microgel pairs using dSTORM (red squares) and from the peak position q_{max} of the scattered light intensity $I(q)$ via $d = 2.2\pi/q_{max}$ (black squares), for details see ref. [2]. The solid line shows the predicted distance assuming $d/2R_{\rm tot} = 1$ for $\zeta = \zeta_J = 0.64$ and decreasing as $d \propto \zeta^{-1/3}$ for $\zeta > 0.64$. The first dashed vertical lines indicates $\zeta = \zeta_c = 0.87$. The second line marks $\zeta = 1.08$ where the brush like repulsive interactions would diverge for particles with a solid core (with core-radius $\alpha \cdot R_{\rm tot}$ ($\alpha = 0.84$)). The measured distances are in agreement with the scenario proposed in the text.

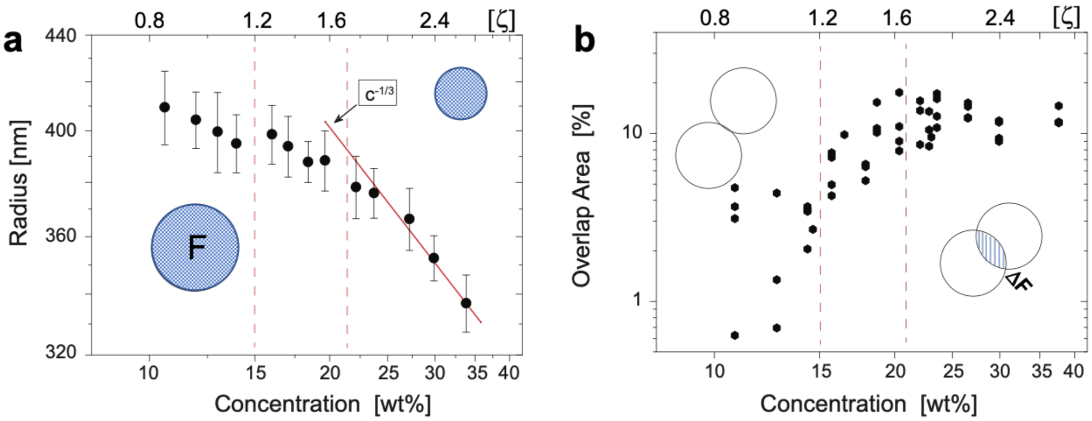

FIG. 5. **Microgel size evolution and interpenetration**. (a) Effective radius extracted from the dSTORM particle surface area F, delineated by the contour line, see also Figure 2 and Supplementary Figures 2 and 9. Solid line indicates the concentration dependence expected for isotropic compression, $c^{-1/3}$, where c denotes the pNIPAM concentration in wt/wt% and $\zeta = k \times c$ ($k = 0.08$). b) Raw data obtained from two-color dSTORM for the relative overlap area $\Delta F/F$ (overlap area Δ divided by the particle area F) as a function of concentration, each point corresponding to a single pair as shown in Fig. 2. Mean value per concentration is shown in Fig. 4 d).

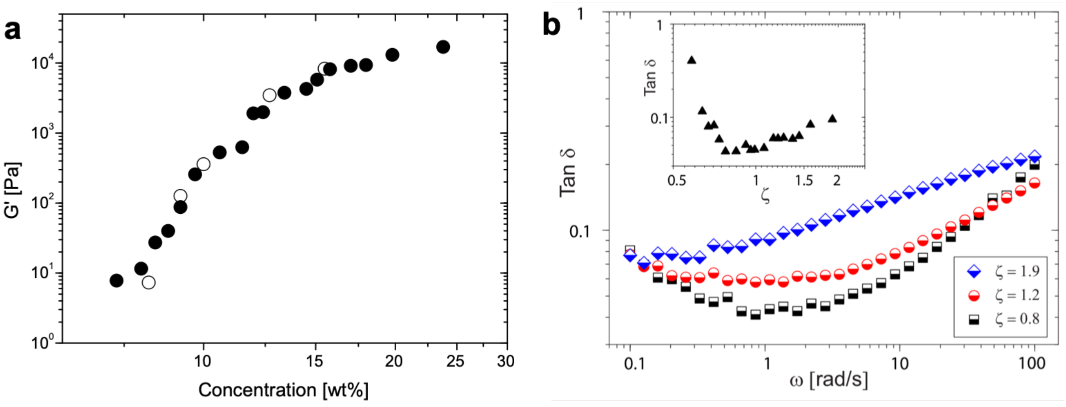

FIG. 6. **Microgel suspension rheology** a) Elastic modulus $G'(\omega = 1.2\text{rad/s})$ as a function of microgel concentration in wt% with and without adding MEA. Measurements performed in pure water (•) and in aqueous solution containing 50 mM MEA (∘). b) Loss tangent $\tan\delta = G''/G'$ as a function of frequency ω for different packing fractions, ranging from marginally jammed to deeply overpacked. Inset: $\tan\delta$ as a function of packing fraction ζ for $\omega = 1.2\text{rad/s}$ (same data as shown in Fig. 4 d)).

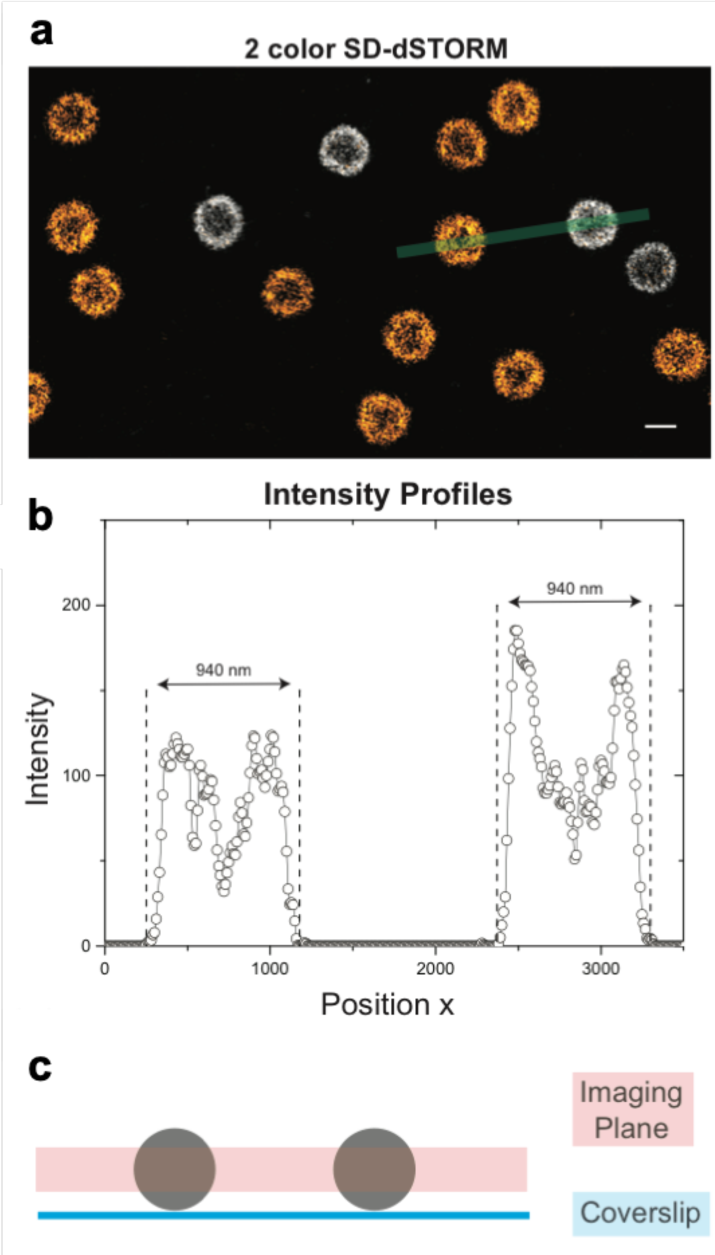

FIG. 7. **Positioning and image acquisition in two-color dSTORM**. a) Two-color spectral demixing (SD) dSTORM image of microgels sparsely deposited on a glass substrate. The example demonstrates the excellent color separation, scale bar 500 nm. (b) Example of intensity profiles of differently labeled microgels, taken along the green line, showing very similar results and sizes. (c) Schematic of how individual microgel particles are imaged when attached to a surface.

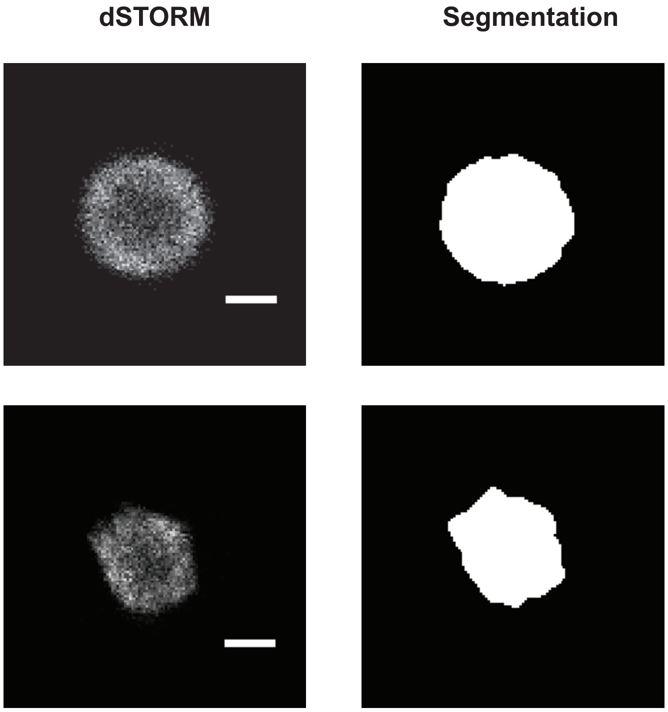

FIG. 8. **Segmentation between particle and background in dSTORM images of microgels**. Two different examples are shown, one image taken for a lower and one for a higher packing density. The images are obtained with the Laplacian of Gaussian edge detection method. The difference between the two is evident but the residual roughness of the contour inhibits a quantitative characterization of shape deformation. Scale bars 300 nm.

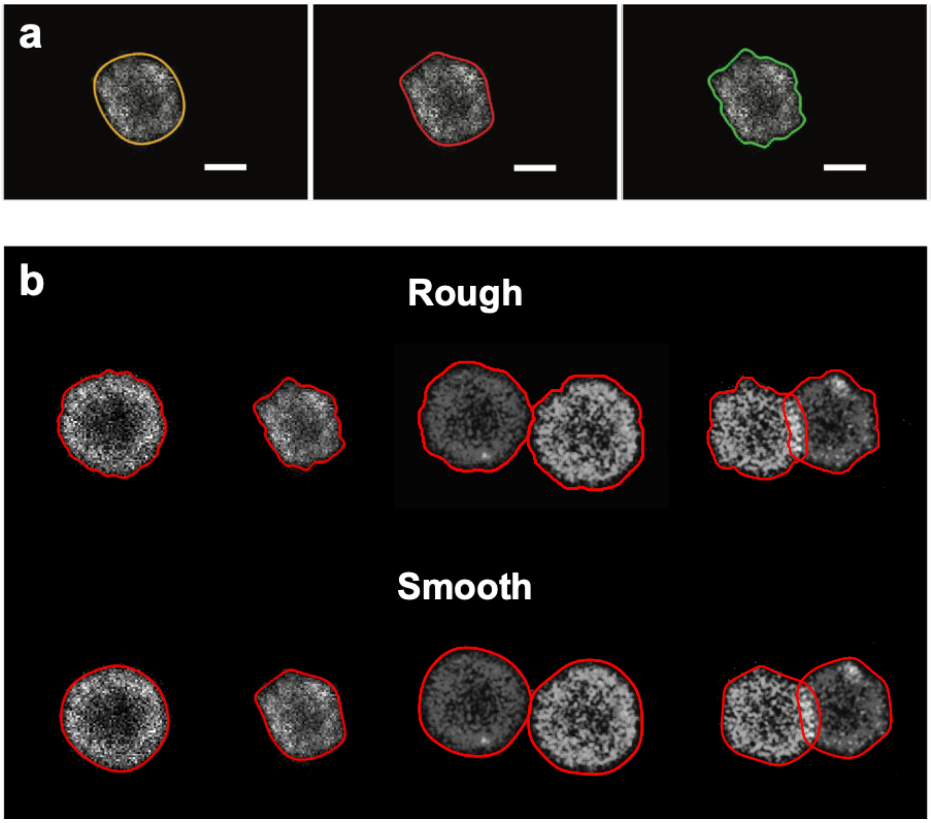

FIG. 9. **Contour smoothing by Fourier descriptors.** a) Lines obtained using different numbers of Fourier descriptors showing progressively more roughness. From left to right $N' = 3$,6 and 30. Scale bars 300 nm. For our particle analysis we use $N' = 6$ which offers the best compromise between a smooth contour and an accurate representation of shape deformations. b) Round and deformed particle contours for single particles imaged with one color dSTORM and for adjacent particle pairs imaged with two color SD-dSTORM, before (N'=30) and after smoothing (N'=6). The example also shows that the overlap area ΔF is basically not affected by the choice of N'.

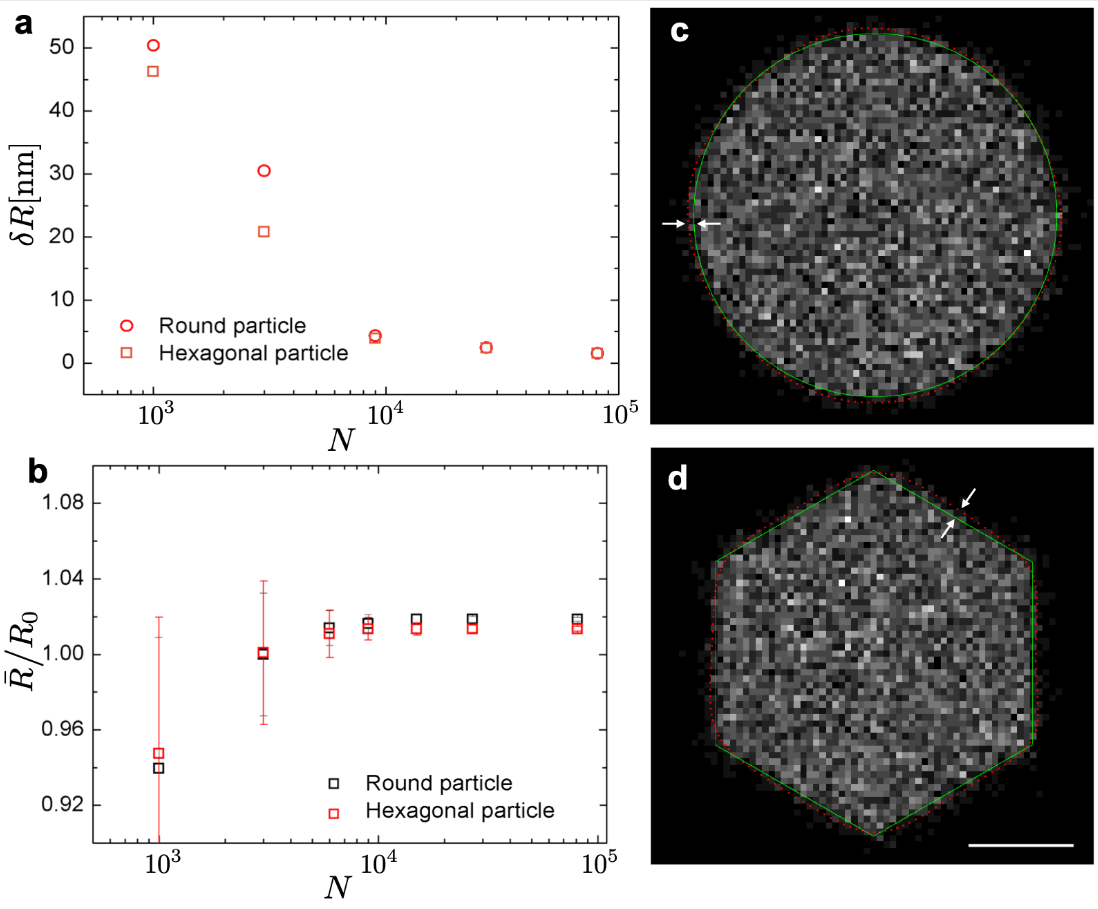

FIG. 10. **Accuracy of edge detection procedure.** Analysis of synthetic dSTORM images of simple geometric objects. The synthetic dSTORM point pattern is created assuming a 30nm optical lateral resolution and then converted to an image with square pixels of edge length 15nm as in the experiments. a) Statistical error when locating the edge of a round (disc shaped) object with radius R_0 = 430nm and a hexagonally shaped object with edge length 430nm. Each data point shows the angular average of the statistical error δR (standard deviation relative to the mean $\bar{R}$) obtained by analyzing the contour lines obtained for 1000 synthetic data sets with identical number of points N. For N >10'000 dSTORM points, as in our experiments, the statistical error is less ±5nm. b) Angular averaged ratio of the contour line position $\bar{R}$ and the reference position R_0. As a reference we take the geometric value R_0 (for the hexagon $R_0(\varphi)$ depends on the polar angle φ which is taken into account). c,d) Typical images for one realization (N = 9000) for each geometry. The red dotted line shows the contour and the green line the geometric shape of the object. The small difference between the contour line and the geometric shape is highlighted with arrows at two positions. Scale bar is 250nm.

SUPPLEMENTARY REFERENCES

[1] Markus Stieger, Walter Richtering, Jan Skov Pedersen, and Peter Lindner, "Small-angle neutron scattering study of structural changes in temperature sensitive microgel colloids," The Journal of Chemical Physics **120**, 6197–6206 (2004).

[2] Gaurasundar M Conley, Philippe Aebischer, Sofi Nöjd, Peter Schurtenberger, and Frank Scheffold, "Jamming and overpacking fuzzy microgels: Deformation, interpenetration, and compression," Science Advances **3**, e1700969 (2017).
